# Narrative as active inference: an integrative account of cognitive and social functions in adaptation

**DOI:** 10.3389/fpsyg.2024.1345480

**Published:** 2024-06-06

**Authors:** Nabil Bouizegarene, Maxwell J. D. Ramstead, Axel Constant, Karl J. Friston, Laurence J. Kirmayer

**Affiliations:** ^1^Department of Psychology, University of Quebec at Montreal, Montreal, QC, Canada; ^2^Research Center, Montreal University Institute of Mental Health, Montreal, QC, Canada; ^3^VERSES Research Lab, Los Angeles, CA, United States; ^4^Wellcome Centre for Human Neuroimaging, University College London, London, United Kingdom; ^5^School of Engineering and Informatics, The University of Sussex, Brighton, United Kingdom; ^6^Culture and Mental Health Research Unit, Lady Davis Institute, Jewish General Hospital, Montreal, QC, Canada; ^7^Division of Social and Transcultural Psychiatry, Department of Psychiatry, McGill University, Montreal, QC, Canada

**Keywords:** narrative, active inference, narrative identity, episodic memory, future projections, storytelling practices, enculturation

## Abstract

While the ubiquity and importance of narratives for human adaptation is widely recognized, there is no integrative framework for understanding the roles of narrative in human adaptation. Research has identified several cognitive and social functions of narratives that are conducive to well-being and adaptation as well as to coordinated social practices and enculturation. In this paper, we characterize the cognitive and social functions of narratives in terms of active inference, to support the claim that one of the main adaptive functions of narrative is to generate more useful (i.e., accurate, parsimonious) predictions for the individual, as well as to coordinate group action (over multiple timescales) through shared predictions about collective behavior. Active inference is a theory that depicts the fundamental tendency of living organisms to adapt by proactively inferring the causes of their sensations (including their own actions). We review narrative research on identity, event segmentation, episodic memory, future projections, storytelling practices, enculturation, and master narratives. We show how this research dovetails with the active inference framework and propose an account of the cognitive and social functions of narrative that emphasizes that narratives are *for the future*—even when they are focused on recollecting or recounting the past. Understanding narratives as cognitive and cultural tools for mutual prediction in social contexts can guide research on narrative in adaptive behavior and psychopathology, based on a parsimonious mechanistic model of some of the basic adaptive functions of narrative.

## 1 Introduction

The ubiquity and importance of narratives has been recognized by many disciplines (Sarbin, 1986; Turner, 1996; Angus and McLeod, 2004; Boyd, 2010; McAdams and McLean, 2013; Taylor, 2016), but an integrative model of the mechanisms linking narrative to human adaptation is lacking. Narratives are reports of real or imagined events, which can be presented in language (verbally or textually) or through sequences of images or other symbols. The narrativity of these reports lies not just in their content, which may point directly to specific events, but also in their structure: that is, the ways in which the linguistic or other symbols convey a set of relationships among events, especially temporal relations (Bal, 2017). In addition, narrative has both social-interactional and cognitive-representational functions (McAdams, 2006; Hutto, 2007; Hutto, 2017); however, the extant literature tends to focus only on one of these two related aspects.

This paper proposes a model of the adaptive mechanisms at play in the production and use of narratives that can account for key aspects of both their cognitive and social functions. We are concerned with the structure of narrative rather than with their content, and as such focus on the implications of the ability to produce narratives rather than what is told through them (Adler et al., 2016). Hence, our account is relevant to understanding structural features of narratives, such as coherence (McAdams, 2006; Adler et al., 2016) and their socio-cultural status as master narratives (McLean and Syed, 2015), rather than with the impact of specific narrative themes (Adler et al., 2016) or fine-grained analysis of modes of narrativity through which experience is given story-form (Abbott, 2014; Bal, 2017).

To motivate our model, we re-examine work approaching narratives from complementary perspectives: as cognitive schemas in personality and developmental psychology (Nelson and Fivush, 2004; Singer, 2004); as cultural tools in social psychology (i.e., master narratives; McLean and Syed, 2015), and as storytelling practices in anthropology and philosophy (e.g., Hutto, 2017). We argue that many of the functions associated with structural aspects of narrative can be understood in terms of the principles of *active inference* (see Table 1 for a summary of the main claims). This perspective shows the complementarity of cognitive and social functions and points the way to novel directions for research.

**Table 1:** Functions of narrative in cognitive and social adaptation from the active inference perspective.

Section	Main claim
3. Narrative as active inference	Narratives can function to create and manage predictions at various levels of representations.
3.1.2 Event narratives as active inference	Memory. Narrative can serve as a means to represent information about past events. They segment specific events, allowing us to build and maintain a pool of information about these events. This pool of information is used to make more useful predictions about the world.
3.1.2 Event narratives as active inference	Episodic future projection. Narratives can play a vital role in episodic future projection. They structure the imagination of the future by integrating past experiences into future scenarios. This function enables us to simulate and anticipate potential future events.
3.2 Narrative identity as active inference	Coherent identity and prediction. Narratives contribute to maintaining integrity in the representations of our experiences. They help minimize internal contradictions and provide expectations of future events and life trajectories. This coherence function ensures that our predictions remain consistent with each other.
3.2 Narrative identity as active inference	Meaning-making and model updating. Narratives facilitate meaning-making by allowing us to draw new connections between different facets of our experiences. They help in incorporating changes in representations (model updating) and assimilating unexpected information.
3.3.2 Socio-cultural narratives as active inference	Sociocultural practices. Narrative storytelling practices can be understood as providing immersive regimes of attention and cultural cues. They prompt individuals to react in culturally prescribed ways and engage with their social and cultural environment. This function is crucial for social coordination and mutual prediction.
3.3.2 Socio-cultural narratives as active inference	Master narratives. Narratives serve to synchronize the predictions that group members generate for their own and others’ future states, behaviors, and relationships. This also makes it easier to predict each others’ behavior.

Active inference is a theory of perception, learning and action, which aims to provide a unified explanation of these basic cognitive functions, as all operating under the same imperative to minimize the difference between what was expected and what was experienced (i.e., prediction error). Originally developed in computational neuroscience as a theory of the structure, function, and dynamics of the brain (Friston, 2010), active inference provides a broad framework that has been extended to explain cognitive systems across multiple scales (Friston, 2013; Ramstead et al., 2021; Parr et al., 2022). Recently, active inference has been applied to the study of social and cultural dynamics in humans, including: the enculturation of agents through immersion in cultural practices (Ramstead et al., 2016), the tendency to conform to social norms and abide by shared values in decision making (Constant et al., 2019), and the capacity for implicit cultural learning (Veissière et al., 2020).

Narrative capacity likely emerged in human evolution as a form of information exchange and knowledge sharing in response to specific adaptive challenges associated with living in groups (Sugiyama, 2001; Boyd, 2018). Narratives offered cognitive and social adaptive advantages for early humans by enabling more efficient storage and transmission of information among members of a group. This included the ability to convey information about the environment, inform others about potential dangers and resources, plan and coordinate collective activities, and pass down knowledge to offspring. The evolution of narrative is a complex process that spans the course of human history from early forms of communication in *Homo erectus* (e.g., gestures, mimetic expressions, and simple vocalizations) to the efflorescence of narrative forms in contemporary societies. Dissanayake (2012) suggested that the first narratives were musical, just as we can exchange information with toddlers using only sounds. The emergence of language (Boyd, 2018) and preliterate poetics (Dissanayake, 2011; Collins, 2014) were pivotal steps that allowed humans to convey more information, as well share more complex experiences, and plan more complex activities. Language enabled the efficient expression of narratives, and narratives in turn provided a context for language development, in a bootstrapping process. Finally, the combination of cognitive predispositions for play, imagination, and linguistic skills allowed for the invention of narratives that extended beyond immediate reality, exploring a broader range of human possibilities through counterfactual, subjunctive, and fictional modes of cognition (Dor, 2017). Narrative practices of storytelling serve multiple social functions, including transmitting survival-relevant information, regulating social relationships, and establishing the identity and social status or position of the speaker (Bietti et al., 2019). The evolutionary approach has been expanded by examining the dynamics of the novel modes and media of narrative at play in our contemporary societies as vehicles for both local and large-scale coordination of groups, and as generators of increasingly complex identities and life trajectories for individuals and communities (e.g., Stubbersfield et al., 2017; Sugiyama, 2019).

In this paper, we contribute to the evolutionary approach to narrative capacity by applying the framework of active inference to outline an integrated theory of the cognitive and social adaptive functions of narrative as concerned with generating and managing predictions. In effect, we argue that narratives are *for the future.* In what follows, we first present a succinct account of active inference. Then, we present our argument through a selective review of the literature on relevant aspects of narratives. We illustrate the utility of the approach with examples. Of course, narratives come in many forms and are used in very diverse ways and detailed consideration of how each of these might benefit from an active inference view is beyond the scope of this paper. Further, we do not believe that active inference can account for all functions and purposes of narrative. We propose an approach to narrative that leverages the resources of active inference to account for some key features of narrative relevant to their evolutionary emergence and adaptive function. An active inference model of narrative can clarify some mechanisms at play in the use of narrative in adaptation and everyday functioning at both individual and group levels. This can guide novel research on narrative inquiry and inference as well as on potential maladaptive consequences of certain forms of narration.

## 2 Active inference

What follows is a brief, non-technical overview of active inference. More extensive and technical presentations of active inference are available (e.g., Parr et al., 2022).

### 2.1 Perception and action

Active inference emerged from *predictive processing*, an umbrella term referring to several related approaches to modeling brain function (e.g., Rao and Ballard, 1999; Clark, 2015). The basic assumption in these approaches is that the brain uses predictive coding algorithms to process sensory information more efficiently. These algorithms are more efficient because they limit information processing to predictions and prediction-errors. Instead of analyzing the entirety of sensory data from the bottom up, the brain uses its best model of the world to predict what the sensations in a forthcoming event will be, and only processes signals that track divergences between expected and actual sensory data. Such models are called ‘generative models’ because they are models of what ‘generates’ the observable sensations from unobservable causes (which are variously called states of affairs in the world, hidden states, latent states, etc.)

The strategy of prediction error minimization can lead to a major economy in information processing because sensations that were accurately predicted need not be further processed. In addition, the prediction errors are used to constantly improve the generative model in an iterative process. Of course, the perfect model can never be obtained, both because we live in dynamic, ever-changing environments and because we never have complete access to our environment through our senses. Thus, the current best model is not the “true” representation of the environment, but the one that yields the least prediction error relative to one’s adaptive goals or necessities.

According to active inference, perception, learning and action selection are the “internal” solutions an agent has for keeping track of and modeling an ever-changing world. Active inference can be viewed as an extension of the predictive coding framework to account for action, using the same processes as those pertaining to perception under predictive processing (Parr et al., 2022). To extend this logic to action, it is posited that predictions are made about body position and an action is a process of matching bodily state to prediction, in a self-fulfilling prophecy of sorts. By incorporating action as a prediction-error minimization process, active inference addresses the problem of evolutionary adaptation to ever-changing environments. On the sensory side of the loop, action is cast as the process whereby the agent proactively and selectively samples sensory data that conform to its expectations about the kinds of outcomes and events that it should encounter, and on the motor side of the loop, the production of movements conform to its expectations serves to further minimize prediction error.

More formally, action, or the selection of one among competing plans or courses of action (which are called *policies* in the active inference literature) is based upon a generative model of the consequences of action (e.g., ‘What will my action lead me to see if I look over there?’). Active inference provides a formalism for the tautology that active agents, like humans, usually select courses of action that lead them to outcomes that they prefer. Perception and learning correspond, respectively, to making inferences about what is causing an agent’s sensory data, and about the parameters of their probabilistic representations of the world. Agents are modelled as acting by selecting courses of action that get them closer to outcomes which they expect, and which are characteristic of “the kind of agent or person that they are.” These two sets of basic processes are important for our account of narrativity: (i) perception and learning, which refers to the error minimizing updates of expectations about states and outcomes of the world, and (ii) action or policy selection, which refer to the proactive sampling of sensory entries that confirm our expectations about “what will happen next.”

### 2.2 From action to culture

Human adaptation, survival, and everyday functioning involve not just individual cognitive agents but coordinated, cooperative action in social groups. Recent applications of active inference to the study of group action in humans focus on the social construction of niches that provide *cultural affordances,* that is, culturally specific possibilities for action (Ramstead et al., 2016; Constant et al., 2019; Veissière et al., 2020; Constant et al., 2022). This work suggests that the social-ecological niche—namely, the set of persons, material structures, institutions, norms, and culturally patterned practices that structure social life for a given group—participates in the error minimization process described above (Constant et al., 2018).

Human beings become encultured, learning the shared expectations that define a given cultural milieu, through immersive participation in specific (socially and culturally constructed) environmental niches (Lende and Downey, 2012). Thus, enculturation occurs through engagement in patterned cultural practices that indicate what is salient and what is not in a given niche—this includes *attention* (Ramstead et al., 2016). These accounts focus on shared patterns of attention and attribution of salience. The hypothesis is that these shared attentional styles result from immersive participation in patterned cultural practices that have the function of enculturating human agents through selective learning. This enculturation via implicit social learning, in turn, is enabled by the involvement of human agents in forms of joint and shared attention and intentional activities (Tomasello, 2014). The socio-cultural niche—as a set of collectively constructed, externalized, and materialized expectations about adaptive and normatively appropriate behaviors—functions as a repository for action-guiding cues. These cues are leveraged by individuals in performing perception, learning and action and, in so doing, help shape or fine-tune synchrony or conformity to the cultural milieu (Constant et al., 2019).

Accordingly, to better infer current states and predict future states (especially those of other group members), humans make use of shared expectations provided by culturally constructed niches. These provide a ready-made set of predictions that are more likely to be accurate because they were tried-and-true by past and current generations. For instance, humans regularly share narratives of the expected life course in their niche (e.g., get a job, get married, buy a house, and have children; Berntsen and Rubin, 2004), which yield expectations that become goals and hence are accurate in a self-fulfilling manner. Shared expectations are also an efficient way to convey the intentions and behaviors of multiple agents (Veissière et al., 2020). In sum, according to active inference models of culture, cultural affordances and shared expectations benefit human agents because they provide information about the everyday behavior of people in one’s cultural and social niche or milieu. This, in turn, is useful because it enables humans to mutually predict each other’s behavior.

Of course, recycling inferences that were useful in the past is not the only strategy used to generate accurate predictions. There is no *a priori,* historical, normative or encompassing set of predictions that can reduce all uncertainty about the future, simply because we live in dynamic environments that are inevitably subject to change. Human adaptation requires exploring these changing environments and learning how to respond adaptively. The ability to integrate novelty is essential to formulate accurate inferences and predictions in a changing environment. Indeed, all personal or cultural predictive models are bound to eventually be updated, refined, or replaced.

Importantly, active inference can also account for the spontaneous and creative processes involved in exploration, novelty-seeking and the generation of new modes of actions (Parr and Friston, 2017). Exploration of new meaning can be cast as serving an underlying conservative principle: that of minimizing unexpectedness and uncertainty. Information gain quantifies the amount of uncertainty resolved by making an observation; and so, it makes sense that humans would seek to resolve uncertainty by actively seeking novel (and information-rich) experiences, which allow us to make longer-term predictions about changing environments.

The ability to integrate novelty is essential to formulating and testing accurate predictions in a changing environment. Exploration and novelty-seeking can be used as pre-emptive strategies to avoid the surprises that will eventually arise. Effective predictions may mostly be repetitions conserved from the past experiences as part of a shared cultural repertoire. However, it is possible for anyone to imagine the future in novel ways and be confronted with surprises, which can lead to more accurate models. As such, exploration is an essential part of our predictive strategies, and narratives are especially useful in such imaginative exploration, entertaining counterfactual situations, binding together sequences of events in long temporal arcs, and extrapolating far into the future (Suddendorf and Corballis, 1997).

## 3 Narratives as active inference

Our thesis is that narratives evolved in part because they provide humans with an improved capacity to proactively, and collectively, formulate, share, and revise their inferences and predictions. Our account was developed, in part, as an attempt to enrich and expand the approach of Hirsch et al. (2013), who proposed that narratives exist at the most integrative hierarchical level of the generative models of agents. We contend that the ability to use narratives (compared with the inability, or reduced ability to do so) enhances the capacity for active inference in human individuals and communities. The main reason for this is that narratives are reports of real or imagined events (or relationships), and the ensuing ability to represent and predict *events* (and their contingencies), instead of only decontextualized elements (e.g., objects, people, places), enriches the repertoire of predictions in a way that serves human adaptation and everyday functioning. More specifically, we will argue that narratives improve the capacity for predictive processing because they (1) can represent a wide array of distinct types of events (e.g., specific past and future events, life stories, and socio-cultural routines and rituals), (2) can represent several events and their interconnections or interdependencies, especially concerning their temporal sequence and causal structures (e.g., linking one’s past with one’s future) and (3) can represent events and their interrelationships in a way that can be easily shared within a large community, as elaborated in the literature on master narratives (e.g., McLean and Syed, 2015) and storytelling practices (e.g., Hutto, 2016; Smith et al., 2017; Bietti et al., 2019).

To be clear, our account does not aim to explain all aspects and functions of narrative. Indeed, narratives have been with humans, in one form or another, since our distant past (Dissanayake, 2012; Boyd, 2018). This basic capacity has been elaborated in ways that have become a very rich and complex facet of our social world, serving a wide variety of functions that active inference does not in itself explain. For instance, narrative can be used as a technology in politics and marketing to persuade others (e.g., Dahlén et al., 2009; De Graaf et al., 2012). They are deployed through rhetoric to gain political power or influence (Brooks, 2022). Narratives are among the most widely available vehicles for entertainment in the world and create aesthetic pleasure in multiple ways. Narratives can be used to self-regulate emotional states (Schaeffer, 1999) and to relieve or prevent stress (Martin et al., 2018).

Active inference can shed light on each of these uses of narrative but does not fully account for them. For example, Kukkonen (2020, 2024) suggests that in reading literature, we engage with the text in ways that are similar to our engagement with the world through predictive processing. Some of the aesthetic qualities of literary narratives and other art forms can be understood in terms of active inference (Van de Cruys et al., 2024). While active inference can model facets of each of these uses, we do not contend that all the functions that narratives serve in our contemporary world fit under the umbrella of active inference. Our account is limited to the ways that narrative help individuals and groups to predict and thereby adapt to a changing environment.

It is also important to recognize that many of the common uses of narrative are not adaptive and these harmful effects may involve processes that are also not fully captured by the active inference account—which may nonetheless explain some of their dynamics. For example, while narratives can contribute to adaptive fitness because they convey knowledge that guides behavior (e.g., Sugiyama, 2001), some of the uses and abuses of contemporary narrative in entertainment are clearly not adaptive (i.e., binge watching, Forte et al., 2021). Dubourg and Baumard (2022) suggest that narrative fictions in entertainment technologies may be akin to ethological superstimuli (Tinbergen, 1969; Barrett, 2010). This process can be cast in active inference terms to capture some of its individual and social dynamics (Ciria et al., 2022).

In the remainder of this paper, we unpack the argument that a key function of narratives in human adaptation and everyday functioning is their use as a tool for inference and predictions and for larger-scale group-level coordination, based on the active inference framework. In particular, we propose a *multi-level* account of narrative in which many cognitive and social functions of narrative can be understood in an active inference model. We proceed by reviewing key aspects of narrative established in the literature, followed by an account of how each aspect can be recast in terms of active inference, thereby providing proposing a cohesive (if necessarily incomplete), testable theory of the functions of narrative as prediction. In the conclusion, we outline empirical and conceptual implications.

### 3.1 Narratives as cognitive schema

In many domains of psychological research, especially personality and developmental psychology (e.g., Nelson and Fivush, 2004; Singer and Blagov, 2004; McAdams, 2006), narratives are understood as a type of cognitive schema, representation, or form of thought instantiated in the brains of individuals, and used to imagine, interpret, reason about, plan, and evaluate actions and situations in their personal lives. These personal narratives have been the focus of two distinct lines of research examining narratives of specific events related to one’s personal life (e.g., Singer and Salovey, 1993), and autobiographical narratives or life stories that organize events and information about personal lives into meaningful themes, developmental arcs, scripts, and self-depictions, thereby increasing the coherence of our identity (McAdams, 2006).

#### 3.1.1 Event narratives: review of theory and research

Narratives contribute to our capacity to represent specific events (past, future, or imaginary) as distinct episodes (Labov and Waletzky, 1967; Nelson and Fivush, 2004; Habermas and Diel, 2013). Experimental evidence suggests that narratives are involved in the parsing of experience into distinct events by determining their beginning and their end (e.g., Speer et al., 2007; Ezzyat and Davachi, 2011). For instance, the narrative binding of experiences into coherent units facilitates their recall (Speer et al., 2007; Wang et al., 2015). Narrative segmentation is central to the representation of specific past events, i.e., to *episodic memory* (Tulving, 1985), and should likewise be central to the representation of future events or *future projections* (D’Argembeau and Van der Linden, 2004).

Episodic memory involves the recollection of distinct, cohesive past events (Tulving, 1985). Some have argued that narratives provide basic structure to episodic memory (e.g., Rubin, 2006) and are important for its development, in large part through their role in the temporal segmentation of information into events with beginnings and ends (Speer et al., 2007; Wang et al., 2015), the arrangement of events on a temporal arc or trajectory (Nelson and Fivush, 2004; Singer, 2004; Reese and Newcombe, 2007; Keven, 2016), and internal relationships that link events by causality or other generative mechanisms that can bridge non-contiguous events (Cohn-Sheehy et al., 2021). This internal predictive or generative structure of narrative allows great efficiency in information storage because the trajectory can be reconstructed from the events and the generative process (Gemici et al., 2017). Although episodic memories are sometimes described as discrete ‘snapshots’ of experience, they are generally reconstructions based on narrative templates (Kirmayer, 1996; Nelson and Fivush, 2004; Rubin, 2006; Wang et al., 2015; Keven, 2016). Indeed, while episodic memories seem to provide the building blocks for narrative accounts, some scholars have argued that all episodic memories can be understood as forms of narrative (Hoerl, 2007). Narratives may bind together more rudimentary forms of event memory to produce human episodic memory (Nelson and Fivush, 2004; Keven, 2016). The importance of this capacity is supported by evidence that individuals who have difficulty providing narratives of their lives using detailed accounts of specific events are more likely to have difficulties in adaptive functioning (Williams, 1996; Angus and McLeod, 2004; Conway, 2005; Dimaggio et al., 2016). Implicit in these accounts is a kind of ‘coarse graining’ into discrete events that scaffold episodic memories.

Future projections involve representations of specific events that could realistically happen to someone in their future (D’Argembeau and Van der Linden, 2004). These projections have been found to share a striking similarity with episodic memories in behavioral and phenomenological characteristics and underlying cognitive processes (D’Argembeau and Van der Linden, 2004), and seem to involve activation in the same or overlapping brain circuits (Schacter et al., 2012). In line with these findings, Schacter and Addis (2007) proposed that an important function of episodic memory is to help individuals create adaptive projections to specific future events and they have provided extensive empirical support for this hypothesis (Schacter and Addis, 2020) This perspective—that memories are essentially *for the future*—has been advanced in anthropology (Young, 1996) and philosophy (De Brigard, 2014) as well. In this view, episodic memories provide a pool of information, based on previous experiences, from which elements can be selected and recombined to create more accurate representations of future events. In the same way that narrative operates to segment past events in terms of beginnings and ends and array them along a trajectory, it may be involved in the segmentation of future events into steps or sequences along a trajectory (Nelson, 2007)—although experimental evidence of this process is still needed.

Episodic memories and future projections are most often operationalized as event narratives (e.g., D’Argembeau et al., 2012). In addition to temporal sequences, there are other processes involved in the formation of episodic memory and future projections, for instance *scene construction* (Hassabis et al., 2007; Race et al., 2011), and causal emplotment (Cohn-Sheehy et al., 2021), to which narratives may contribute. Through temporal structure, scenario building and emplotment, narratives are centrally involved in the recollection and representation of specific past and future episodes and events.

#### 3.1.2 Event narratives as active inference

We can appeal to active inference to explain the efficacy of narratives of specific past and future events. As noted above, a recent perspective on memory and future projection argues that the adaptive function of future projections is increased by the incorporation of information provided by memories of specific past events. This constructive episodic simulation hypothesis suggests that the basic adaptive function of episodic memories is to guide, inform, structure, and constrain the representation of future events (Schacter and Addis, 2007, 2020). The idea that reliance on past event narratives can increase the accuracy of predictions and the adaptive value of future projections has received much empirical support (e.g., Suddendorf and Corballis, 1997; Wheeler et al., 1997; Atance and O'Neill, 2001; Klein et al., 2002; De Brigard, 2014; Conway et al., 2016). Narratives are crucial for this process in that they organize past and future experience into units, connect them in sequences, and tag them in ways that make them accessible in situations in which similar cues or sequences of events occur.

Under active inference, a function of specific past event narratives can be understood as providing a pool of information made of past specific episodes, within which regularities can be extracted and leveraged to predict situations which are similar in certain respects to relevant remembered episodes, thereby avoiding the cognitive burden of excessive updating of one’s probabilistic expectations (Schank, 1980, 1990). Agents can draw from a pool of available possibilities that constitute the legacy of personal experience as well as the vicarious experience inherited from others that is encoded in shared cultural narratives. As noted above, individuals who have difficulty recounting rich narratives of their lives using detailed accounts of specific events tend to have difficulties in adaptive functioning (e.g., Williams et al., 1996). Furthermore, because the future is never simply a repetition of the past, multiple past events usually need to be combined to generate accurate inferences about a specific upcoming event (Schacter and Addis, 2007). Even repeated events that have a similar structure over time (e.g., holidays at the grandparents, weekly basketball practice) may have a substantial degree of novelty that can be addressed by incorporating information from other episodic memories or schemas (Conway and Pleydell-Pearce, 2000).

A recent line of research on memory provides evidence consistent with this proposal. Congleton and Berntsen (2020, 2022) have shown that experiencing surprising stimuli tends to increase memory for the event that preceded the surprise. In other words, the novelty of the situation increases the salience of recent events. This functional increase of memory for the context of surprising information was found in one recent study to involve hippocampal activity, which may be an important contributor to updating memory in the case of prediction error (Sinclair et al., 2021).

Taken together, these results are consistent with, and provide at least indirect evidence for, our contention that narratives partly function to implement active inference. Specifically, we propose that narratives of specific past events function as a pool of information aimed at guiding the generation of effective predictions. Well-predicted events (i.e., routines) quickly become forgotten (Conway and Pleydell-Pearce, 2000) because they do not contribute relevant information for predictions (Philippe, 2022). When prediction-errors arise, they lead to the encoding of new memories, because they carry highly relevant information to improve the accuracy of predictions in the future. The formation of new memories, which necessarily involves narratives (Rubin, 2006), can be understood as a process of model updating. Active inference at the level of event narratives involves a *conservative* process of replicating elements of past events into future events that are closely similar to those past events (e.g., habits) which maintains coherence and continuity between past and future, and a *creative* or constructive process that involves the flexible recombination of past events into future events (Schacter and Addis, 2007). Narratives are particularly useful for active inference because they can contribute to both the conservative processes that maintain old memories and use them as models for new events, as well as generating new models through their recombination or projection to produce new stories (Mar and Oatley, 2008).

In summary, event narratives are ideally suited for predictive processing because of their ability to represent past and future events in segmented event sequences, to provide structured information about the past (episodic memories) that can help predict future events, and to project these inferences in the form of cohesive imagined scenarios of future events (future projections). This occurs, first, through an expectation-confirming conservative process, a strategy for prediction that involves a replication of the aspects of future events that should be identical or similar enough to the past, and second, through an expectation-updating, constructive process that involves recombining elements of multiple past events (Schacter and Addis, 2007) or reinterpreting memories in *counterfactual thinking* to update and create new expectations for the future (De Brigard and Parikh, 2019). In the active inference approach, these two distinct functions of narrative are integrated into a single predictive mechanism that aims to find the optimal balance between conservation and change, which is necessary to adapt to an environment that evinces both continuity and change. This balance instantiates aspects of Piaget’s broader notions of cognitive assimilation and accommodation (Piaget, 1976; Block, 1982; Hanfstingl et al., 2022) and provides a way to understand the coordination of these complementary and interdependent processes.

#### 3.1.3 Narrative identity: review of theory and research

A second key line of research on narrative schemas involve the broader depictions of one’s life themes, chapters, and recurrent events, ultimately giving rise to what has been called *narrative identity* (McAdams, 1988, 2018). McAdams (1988, 2018) has argued that identity can be conceived as an ever-evolving internalized global narrative that situates the self in time by broadly reconstructing its past and imagining its future. Narrative identity functions to bring meaning and coherence to one’s life (McAdams, 1995, 2001; McLean et al., 2007). Research suggests a moderate positive association between narrative meaning making and coherence with psychological well-being (Adler et al., 2015, 2016; Habermas and Köber, 2015; Mason et al., 2019). The mechanism explaining this positive relationship is postulated to be that a coherent identity provides a sense of personal continuity and identity integration (Syed and McLean, 2016).

In the context of autobiographical narratives, ‘meaning’ consists of the relations we draw between salient aspects about our experience (e.g., people, places, events, concepts) (Baumeister, 1991; Heine et al., 2006; Park, 2010; Proulx and Inzlicht, 2012). Meaning, on this account, resides in relationships between simple aspects of our experience (e.g., an object and a smell) or complex ones (e.g., an emotion and a familiar location), that often entail a network of associations among multiple elements. Heine et al. (2006) add that meaning concerns the iterative construction and restoring of *expected relations* between relevant information that humans construct about aspects of the world, and as such, it entails establishing, maintaining, and restoring a sense of familiarity (or predictability) with the world. Of course, simply linking salient elements of our experiences does not necessarily result in the construction of coherent ensembles. This requires overarching structures like self-schemas or identity that confer consistency and coherence in a top-down fashion. Another key function of narrative is to integrate our various representations of self with the multiple past and future life events in ways that provide a coherent identity (McAdams, 1995).

Many researchers have explored the possibility that narratives can be used to help support the meaning-making processes centered on the self (Habermas and Bluck, 2000; Singer, 2004). Singer and Bluck (2001) suggest that two processes occur sequentially to facilitate the construction of meaning. First, individuals engage in *narrative processing* of their life experiences, a type of information-processing that organizes information into an event unit by transforming it as a story. The resulting storied accounts of life events open possibilities for further meaning making by subsequently using these narratives of the events of our lives for self-reflection and finding narrative links or associations among them. Once information about our lives is organized in the form of storied past events, it becomes easier to reflect on and link to more, and more varied, aspects of experience by narrating them in relation to the self. This *autobiographical reasoning* (Habermas and Bluck, 2000; Staudinger, 2001) involves drawing on past experiences to explore linkages to other past events, current concerns and actions, and future objectives. This process can lead to new interpretations of past events, and new knowledge about the self (Staudinger, 2001), which may help to predict novel future events. Insofar as the meaning making process involves the creation and maintenance of links between disparate aspects of our experience, a coherent self or identity can be seen as a culmination of this narrative process. It should be noted that the relationship between narrative and meaning-making is generally bi-directional, such that a circular process is at play in which basic forms of embodied and enacted meaning-making underlie narratives and narratives in turn provide a scaffolding for more elaborate meaning making (Singer, 2004).

Narratives can be used to foster consistency among self-related expectations that are temporally recent and distant (Berntsen and Rubin, 2004), grounding the ever-evolving facets of identity in accounts of the personal past and extrapolating them forward in a life trajectory that links stories of origins with current circumstances, and future aspirations or possible selves (Carver and Scheier, 2001; McAdams, 2001; Conway, 2005; Erikson, 2007). This process can integrate temporally local or focal narratives into longer accounts. Longer narratives can lend coherence to shorter narratives by providing a context or frame in which different sub-stories can coexist.

In sum, narratives function to establish expected relations in an iterative process with different phases. First, narratives are involved in stabilizing established cognitive expectations, which is part of the coherence-making function of narrative. Then, narratives are involved in the posterior explanation and interpretation of unexpected or surprising events, updating schemas so that events can be expected in the future (e.g., Habermas and Köber, 2015). Narratives are thus centrally involved in two distinct phases in the process of meaning-making (Park, 2010).

Narrative serves to construct and maintain a coherent identity. A coherent narrative identity is characterized by the fact that it connects past experiences and future plans in ways that can be understood by others in one’s local social world. Narrative coherence is not simply a matter of logical consistency or non-contradiction but follows cultural templates that incorporate local norms and modes of explanation, including the temporal sequences of causality (Howard, 1991). A narrative is deemed coherent, in this sense, when it can be understood in a specific social context (McAdams, 2006), in relation to particular modes of culturally grounded personal interpretation and sense-making (Habermas and Reese, 2015). The coherent grounding of the future in the past also helps to ensure the maintenance of goal pursuit (Conway et al., 2004). In this sense, narrative identity can help to maintain oneself within one’s *viability sets* – the modes of adaptation that are conducive to continued existence —which include environmental states, social niches, and social roles (Di Paolo and Thompson, 2014).

In summary, narrative identity processes provide individuals with the means to efficiently produce, maintain, and change the global relationships among the different facets of their experience and identity: this includes creative processes of narrative construction that generate new facets and configurations of identity, and conservative processes of narrative recollection and retelling that reproduce and stabilize these facets in an internally coherent account of events pertinent to self, personhood and biography.

### 3.2 Narrative identity as active inference

**Figure 1:**
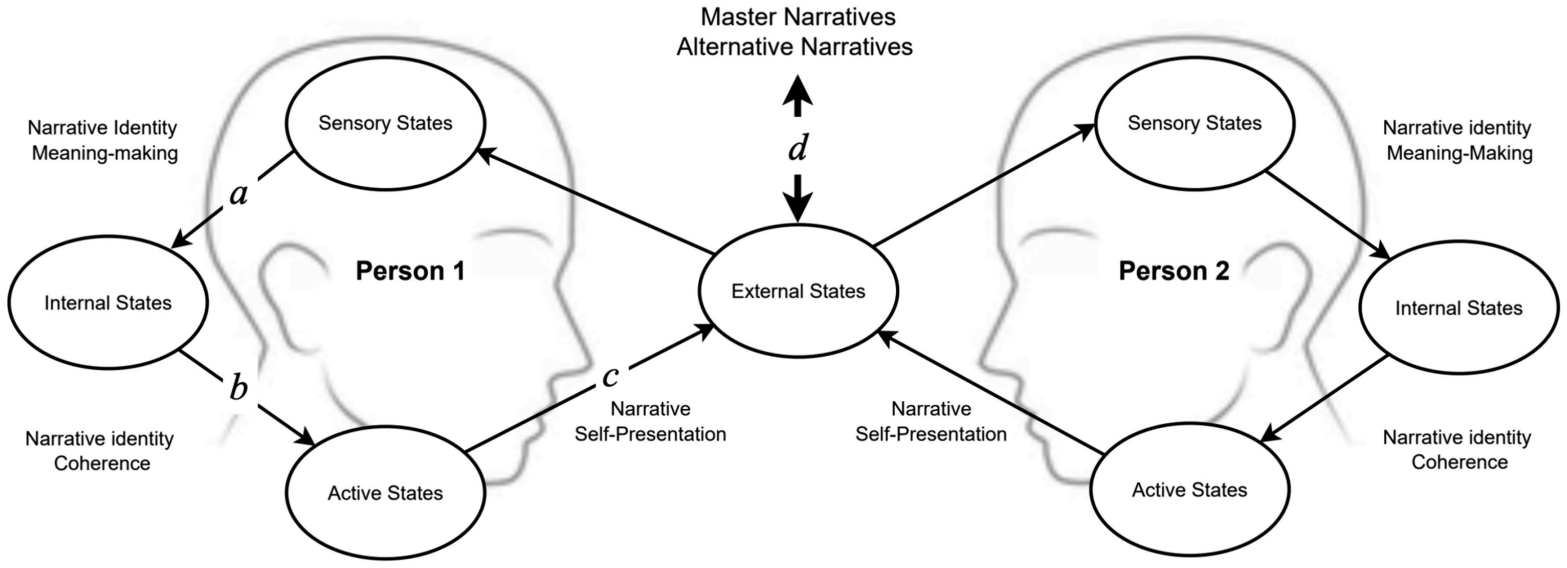
The framework of active inference allows us to combine different conceptions of narratives under one set of dynamics that we here depict in non-technical terms (for a technical description of these dynamics in the context of similar agents-environment interaction dynamics, see: Veissière et al., 2020; Constant et al., 2022). The model depicted in the figure corresponds to the iterative process through which narratives function to establish and engage with expectations through the construction of a coherent narrative identity, social self-presentation, and engagement with cultural master narratives. The model is of a Partially Observable Markov Decision Process (POMDP) under active inference which follows several iterative steps: (a) Narratives are involved in the explanation and interpretation of unexpected or surprising events, updating schemas so that these new events can be predicted in the future; (b) Narratives are used to foster consistency among specific event predictions to yield a coherent *narrative identity* and plan coherent action. The coherent narrative identity enables the person to generate actions that are consistent with each other; (c) Narrative self-presentation refers to how individuals present themselves to others through the narratives they construct about their own lives. This involves the ways that people tell stories about themselves, their experiences, beliefs, values, and identity, to the external world; (d) the actions of each person change the structure of the external world and thereby contribute to the formation of social practices and deontic cues, which may constitute a form of master narrative or provide alternative narrative templates.

Research has linked narrative identity coherence and meaningfulness with adaptive functioning (Adler, 2012; Adler et al., 2015, 2016; Mason et al., 2019; McLean et al., 2020b). This link is generally explained by the fact that narrative identity coherence and meaning give rise to a sense of personal continuity and identity integration (McLean and Syed, 2015). This adaptive value can be understood in part because coherent and meaningful identity narratives are conducive to improved predictive processing; that is, they better scaffold the generative model of the person in the world and the social world and thus provide a way to generate more accurate predictions to effectively navigate socially constituted and encultured worlds (see Figure 1). Identity coherence and meaningfulness also serve to organize personal and social actions in a more predictable way for ourselves (i.e., making one’s own behavior more predictable to oneself) as well as for others (making one’s behavior more predictable to others). Both features of identity can contribute to adaptation: individually, because if we are more predictable to ourselves (e.g., if we have a clearer identity and long-term goals), we can more readily meet our own expectations and achieve our goals; and socially, because if we are more predictable to others, they may find us easier to cooperate with and get along.

First, coherent narratives link aspects of experience together in ways that decrease the contradictions among choices, values, and goals. Coherent identity narratives can thus reduce dissonance among the more complex and over-arching expectations we have about our lives, thus minimizing complexity and keeping things simple by acting like heuristics for cognition (Gigerenzer and Gaissmaier, 2011) that concerns the individual’s global life course. A coherent narrative identity functions to reduce uncertainty about future states because it provides predictions about the events that are likely to occur in a ‘normal’ (i.e., culturally sanctioned and expectable) life trajectory over the course of development (Habermas and Bluck, 2000; Berntsen and Rubin, 2004). Narrative identity can also provide a set of consistent expectations with regard to an individuals’ idiosyncratic goals and life trajectory, which can help the individual be predictable to both himself and others. In contrast, vaguely determined or contradictory global identity narratives provide blurred, contradictory, and imprecise expectations, which entail a greater degree of uncertainty that may increase the cognitive demands or burden of predictive processing and, by extension, impede adaptive functioning. This can occur, for instance, by generating prediction-error when the model fails to predict what will happen next. This effect of increasing prediction-error could partly explain why a coherent narrative identity is positively related to well-being, and why a diffuse or vague identity is a well-known correlate of serious psychological and behavioral problems (e.g., Dimaggio et al., 2016).

The function of narratives as a basis of the meaning making process can also be seen through the lens of active inference. Meaning making involves creating and exploring new links between elements of our experiences, which generates change in representations (McLean et al., 2020b). In a similar vein, Staudinger (2001) proposed that narrative has epistemic functions, in that narratives are used to actively reflect on and evaluate events to identify and integrate new knowledge about the self. This process can be seen as important for the exploratory phase of active inference, also known as *epistemic foraging* (Parr and Friston, 2017), where surprising or new facets of environments are encountered and new, updated models of future events need to be constructed. The meaning-making process is seen in the literature as an adaptive response to the occurrence of unexpected circumstances, when expectations are not met, and an imaginative reorganization of our representations of ourselves and our environments is needed (Heine et al., 2006; Park, 2010; Proulx and Inzlicht, 2012). As such, the meaning making process that is at play in narrative self-fashioning can be cast as active inference insofar as it involves resolving uncertainty by ascribing new meaning to unfamiliar, ambiguous, or disruptive events and integrating them with the known, familiar aspects of our experience to update what is now to be expected in the future.

There is evidence that the meaning-making process is especially associated with well-being when participants have recently experienced unexpected events that challenge their sense of identity (Habermas and Köber, 2015). Consistent with our proposal, this suggests that it is because meaning making restores our sense of narrative coherence and continuity, thereby increasing one’s predictability for oneself and for others. While the most common explanation for the positive relationship between narrative coherence and well-being is that coherence provides a sense of continuity and integration (Pasupathi et al., 2007; McLean and Syed, 2015), our account provides a complementary explanation of the mechanisms at play. First, the association between integrative identity and well-being follows from the way that an integrative identity provides a set of predictions that is more efficient because it contains fewer contradictions, leading to less prediction-error, which thereby improves predictive processing. Secondly, our active inference model of narrativity is consistent with the possibility that a coherent narrative identity helps foster the sense of being the same person across time (i.e., personal continuity). However, it provides a more precise context to understand why this sense of continuity is associated with well-being; namely, that a coherent narrative identity increases well-being and adaptive functioning because it offers a stable and consistent framework from which agents can project themselves into the future with more accurate predictions, which also helps to maintain a sense that the past identity is related to the future identity. Thus, we suggest that a key factor leading from narrative coherence to increased well-being is the fact that a coherent narrative leads to improved predictive processing capacity in individuals.

This is consistent with work by Pasupathi et al. (2007) who describe the role of self-event connections in maintaining a sense of identity through stability and change. They frame identity as a developmental problem, especially in adolescence, and note the challenge of maintaining a sense of personal continuity during the inevitable changes induced by development. The argument suggests that the task of constructing a sense of personal continuity through the inevitable changes is concerned with integrating the past with the present, that is, to “construct both a stable sense of self as well as a sense of how the self *has changed* over time” (Pasupathi et al., 2007, p. 104, emphasis added). They also suggest that the continuity of identity needs to be resolved before it is possible to commit to future selves. Finally, past events are key in constructing the sense of self and identity. Again, an active inference reading suggests that a diametrically opposed emphasis is warranted to really understand the dynamics at play. From the point of view of active inference, personal continuity is especially concerned with how one *will* remain the same over time in the future. An active inference view of narrative identity suggests that creating narratives of the future is a key process in organizing the self. A crucial purpose of building a narrative identity is to orient ourselves towards the future, to predict it and adapt to it. Of course, narratives are used to make connections between the self and the past, as the past contains information necessary to construct and make sense of identity; however, identity is not simply built from the ‘ground up’ from memory to project the self into the future. Expectations of the future (as well as aspirations, goals or plans) constrain what information about the past is relevant to identity. As such, identity continuity can be the result of a narrative built in two directions: from the present to the future and to the past, each of which can modify the other. From an adaptive point of view, much more than historians of our own lives, we are first and foremost actors in an unfolding story that we extend into the future.

Relatedly, another potential role of narrative coherence in active inference may lie in its ability to improve ones’ predictability to others (McLean, 2019); that is, having a coherent narrative identity, displaying a continuous, predictable, stable (narrative) identity might be valued by others who find one more predictable, reliable, and consistent (hence, easy to cooperate with) or because it serves to maintain specific valued traits or roles.

Finally, we must clarify a crucial point. While we have argued that coherence and meaning (which are structural elements of narratives) facilitate active inference and adaptation, they may not always lead to psychological well-being, because it is not the mere fact of having a narrative of identity but also the content of such narratives that matters for psychological well-being. A coherent and meaningful set of narratives optimally reduces uncertainty and facilitates active inference by providing consistent expectations of various future events. However, this is also true of coherent and meaningful narratives with negative content that fuel negative expectations (e.g., failure, rejection, disappointment, inefficacy), which might in some contexts represent the most probable events or, in other contexts, impede adaptation and even sabotage favorable circumstances. Such negative narratives may lead to psychological distress, as is seen for example in the way that negative self-statements and expectations contribute to low self-esteem, depression (hopelessness) and anxiety (Beck, 1979). Indeed, research has shown that narratives with positive and negative content are, respectively, associated with psychological well-being and ill-being (McLean and Lilgendahl, 2008; Lilgendahl and McAdams, 2011; Banks and Salmon, 2013; Lilgendahl and McLean, 2020). However, they may still be beneficial in so far as they provide a means to enhance predictive accuracy. This is consistent with recent active inference models of the etiological pathway from allostatic load to depression that highlight the possibility of acquiring maladaptive expectations when faced with environments that chronically thwart one’s attempts to act effectively in the social world, resulting in learned helplessness (Arnaldo et al., 2022).

The active inference account can also help us understand the remarkable resistance to change of some negative narratives (e.g., Singer, 2019). This inertia suggests that there are processes that maintain negative narratives, despite the distress they cause. From the point of view of active inference, what one gains from a negative narrative is simply the ability to reduce prediction error and prevent future (unpleasant, unfamiliar, dangerous, and ego-dystonic) surprises. Negative narratives can provide accurate expectancies with respect to current and future negative circumstances. Unfortunately, this gain in coherence and prediction may also have maladaptive consequences when the negative narratives are the source of a broader negative sense of self. In effect, the negative expectation may set up a self-fulfilling prophecy that maintains itself through accurate prediction (and production) of negative outcomes.

This raises an important set of empirical questions regarding the adaptive value and consequences of negative narratives. From the perspective of active inference, it should be better to hold a more accurate model of the world, even if it is negative in its evaluation of the self, rather than an inaccurate but more positive model, because it keeps one adapted to the environment (i.e., generates a flow of expectations that reduce prediction error) despite the associated psychological ill-being resulting from pessimism, negative self-views, and so on.

Such maladaptive consequences or trade-offs in active inference could account for other important clinical phenomena. For example, some researchers have observed that victims of childhood abuse tend to re-enact trauma later in their lives, which can result in revictimization (Van der Kolk et al., 1994; Gladstone et al., 2004). The mechanisms underlying this re-enactment are still the object of debate, and explanations have invoked notions of dissociation, complex motivational theories, and even “addiction to trauma” (Levy, 2000). A straightforward account can be given in terms of active inference (Linson and Friston, 2019; Linson et al., 2020). Quite simply, trauma victims expect and enact negative narratives because, given their past experiences, these are the most plausible, coherent, and meaningful possibilities—i.e., those that reduce prediction-error the most (Holmes, 2020). These embodied and enacted expectations are elaborated through the overarching process of establishing a sense of personal continuity and integration by creating, maintaining, and enacting coherent and meaningful narratives. The narratives then guide the interpretation of novel situations in ways that may impede learning new patterns of interaction and adaptive expectations. Of course, nonverbal, embodied expectations also influence interpersonal interactions and may help maintain maladaptive narratives, but the narrative component may be particularly amenable to therapeutic challenge and change.

In summary, we propose that the involvement of identity narratives in individual active inference involves two processes. First, identity narratives lead to the creation and conservation of global coherence among representations in a process that can be seen as a form of active inference about the person in their local world. Second, identity narratives allow the integration of new possibilities to eventually restore a renewed sense of self-continuity and familiarity, to restore a coherent set of expectations and integrate environmental or situational changes. However, the (active inference based) pull towards coherence and meaningful narratives does not preclude the possibility of negative narrative generalization. In fact, it can partly explain why anxious, depressed, or traumatized individuals hold on to negative narrative expectancies so firmly. An understanding of this process of inertia or resistance to change in terms of active inference could be leveraged to develop targeted therapeutic interventions (Linson and Friston, 2019).

#### 3.2.1 Socio-cultural narratives: review of theory and research

In addition to their role in cognition, identity, and memory, narratives have crucial social functions that depend on culture and context. More than just residing in individuals, narratives also reside in cycles of narration – they are constituted through communicative action and hence are not only located in the brain but in embodied loops with local socio-cultural environments (Di Paolo et al., 2018), or cultural affordances (Ramstead et al., 2016). In this section, we review two distinct approaches that understand narratives as social practices or enactments: the Narrative Practice Hypothesis (Hutto, 2016) and the master narrative framework (MNF; Hammack, 2008; McLean and Syed, 2015) to illustrate how an active inference approach to narratives can integrate the internalist cognitive account with a more externalist focus on social practices of narration.

The Narrative Practice Hypothesis (NPH) argues that storytelling and other ways of giving narrative accounts of experience (to oneself and to others) are fundamentally social practices (Hutto, 2012). In this framework, narratives derive their meaning from particular social contexts and have instrumental functions: generating or conveying expectations, regulating self-presentation, negotiating relationships, and generally serving to position the narrator vis-à-vis others in the environment or social system (Gallagher and Hutto, 2008; Hutto and Kirchhoff, 2015; Hutto, 2016).

The NPH suggests that human agents learn to explain and justify themselves (to others and to themselves) by telling stories about why they and others do what they do. This includes *the ability to give reasons for one’s own and others’ actions*. According to the NPH, human agents learn to provide reasons for their own and others’ actions over the course of their development, mostly by participating in a special kind of narrative practice, namely, *folk-psychological narrative practices* (Hutto, 2012). Folk psychological narratives are stories that center on accounts of the mental states of other persons as explanations, causes or outcomes of action. Many (indeed, perhaps most) types or genres of stories (e.g., fairy tales and myths) that adults tell children over the span of development involve the mental states of characters involved. Learning to use and understand these narratives requires that children learn how to make (sometimes explicit, mostly implicit) inferences about agents’ reasons and motives (Taylor, 2012; Taylor, 2016).

The narratives that are produced in acts of storytelling, insofar as they are tangible cultural artefacts, can be seen as anchors of joint and shared forms of attention (Tomasello, 2014; Hutto and Kirchhoff, 2015). The act of storytelling itself occurs in specific social settings and in socially prescribed forms that convey information above and beyond the particular stories being exchanged. Stories and storytelling provide structures and ways of organizing experience and of reading the environment in terms of cultural affordances (Ramstead et al., 2016). In telling and listening to stories, both storyteller and listener are not merely focusing on the story as such, but also on the elements, relationships and narrative structures within the story and their relationships to the larger context of the telling. In doing so, all participants become more predictable to each other.

According to the NPH, ‘folk psychological stories’ or ‘person stories’ provide children with developmental anchors through which they acquire the ability to provide reasons for the way they (and others) acted. In the cultural practice of everyday storytelling, agents participate in narrating ordinary events and experiences of self and others. Participation in such practices leads agents to acquire the folk psychological skills necessary for more elaborate cognition through self-narration and self-presentation. These claims of the NPH are consonant with, and find support in, empirical work on narrative in developmental and social psychology. For example, based on observational, longitudinal, and experimental studies, Nelson and Fivush (2004) advanced a social-cultural developmental theory of the origins of narrative skills and autobiographical memories. They argue that these two capacities (narration and the capacity for autobiographical thinking) emerge in large part as the result of the cultural scaffolding provided by caregivers. More specifically, they contend that the involvement of the child in social interactions, in which the caregiver recounts narratives about shared past events to the child, is crucial for the child’s development of the capacity to use narrative structure and content in cognition, and eventually in autobiographical memories. In doing so, the adult provides the child with a format and organization in terms of which past events can be efficiently encoded and remembered. The structure and characteristics of these storytelling interactions thus give rise to important features of the content, structure, and organization of autobiographical memory. Storytelling interactions vary greatly among individuals and across cultures. Developmentally, everyday storytelling is shaped by characteristics of both parent and child as well as family and larger social contexts (Sahin-Acar and Leichtman, 2020). This variability in conversation gives rise to differences in the number, types, emotional quality, and narrative coherence of remembered events. Further, conversations about shared past events are constrained by sociocultural views about what characteristics of events are salient, worth discussing, and worth remembering (Nelson and Fivush, 2004). For instance, studies have found that mothers who narrated past experiences to their children using more orienting information (i.e., when and where), asking questions and providing hints and details about the recounted event, had children who later reconstructed more coherent narratives that included this information (Haden et al., 1997). This cultural variability suggests that there will be differences in the developmental course of narrative capacities and autobiographical memory across cultures (Howard, 1991). Research has demonstrated that cultures provide different master narratives and different ideas about what makes a “good story” and this influences the ways that individuals narrate their lives (Fivush et al., 2011), articulate social roles and facets of identity like gender (McLean et al., 2020a), and extends to the ways that individuals maintain a sense of continuity of identity (Becker et al., 2018).

The *Master Narrative Framework* (MNF) is another important approach to understanding our embeddedness in culture through narrative (Hammack, 2008; McLean and Syed, 2015). While similar in some respects to the NPH, the MNF is less concerned with how cultural narratives guide actions in specific contexts than with their implication in forming identity representations, focusing especially on the interrelations between cultural and agentic influences. Since master narratives have been inconsistently defined in the literature (Hammack, 2008), we adopt the framework of McLean and Syed (2015), which proposes a more precise characterization of master narratives according to five principles. First, master narratives have many uses for members of a group (*utility principle*), such as providing relevant information to understanding the group’s rules and characteristics (e.g., its history, values, social norms). As such, master narratives facilitate belonging and social integration through the synchronization of referents across group members. They also support personal identity development, by serving as a source of information upon which group members draw to define their identity, as well as to understand the identity of others. Relatedly, master narratives provide information about the typical life course that is expected of a cultural group’s members. Secondly, master narratives are shared by the majority of the members of a culture (*ubiquity principle*), because they are only useful for social cohesion to the extent that they are widely adopted. Third, most members of a culture effectively accept and internalize master narratives without recognizing them explicitly as such (*invisibility principle*). The fact that master narratives are unconsciously internalized as ready-made guides for how to live a life and understand the lives of others facilitates adaptation by alleviating the necessity for effortful reflection on everyday choices, actions or events. The fourth principle refers to the fact that master narratives are not value-neutral from an ethical standpoint; that is, master narratives reflect social norms and expectations. Adopting master narratives may be enforced by social pressure and associated with moral approval or disapproval (*compulsory principle*). As such, master narratives tell us how to avoid criticism, conform to social expectations, and be accepted as ‘good’ members of the group. Finally, master narrative are hard to change (*rigidity principle*), because they are woven into many aspects of the social system, including structures, institutions and routines, but also because those who benefit the most from master narratives are also in power, and use their influence to maintain and enforce the master narratives.

In accord with the principles described above, individuals will for the most part assimilate the master narrative’s ready-made life-course depiction into their personal narratives, usually without being explicitly aware of using it as a model or template. However, because we live in dynamic environments, master narratives eventually become outmoded or obsolete, and alternative narratives are needed for adaptation. Ironically, master narratives may become conscious at such moments of culture change and for individuals who come to reject the master narrative to adopt (and sometimes invent) alternative narratives.

Finally, although the central role of narrative in constructing the self is widely acknowledged, some have argued against its importance (e.g., Strawson, 2004). It seems likely that there are facets of the self that do not depend on narrativity, and these may be developmentally prior, and in some sense, more basic structures on which the narrative self can be scaffolded (Gallagher, 2000). However, there is much empirical evidence that narrative self-construals and organizations of autobiographical memory play important roles in adaptation and, while initially acquired in childhood, these self-narratives continue to change in response to life challenges and transitions (McAdams and McLean, 2013; Fivush, 2022).

#### 3.2.2 Socio-cultural narratives as active inference

What distinguishes human beings from other animals is the temporal scale, scope, complexity, flexibility and inventiveness of cooperative activity, guided by cultural history and practice to fashion a niche constituted of social practices, norms and institutions (Veissière et al., 2020; Henrich and Muthukrishna, 2021).

Storytelling has been an important social process in human cultural co-evolution (Boyd, 2010; Smith et al., 2017). We suggest that *narrative practices* can be understood under active inference in terms of these uniquely human forms of extended phenotype. Integrating the NPH, we suggest that narrative storytelling practices can be viewed through the lens of active inference as *socially shared patterns* that prompt human beings to act in culturally prescribed ways—provided, of course, that they are enculturated in contextually appropriate ways to engage in specialized forms of niche construction through coordinated cooperative action that are specific to the human adaptation and everyday functioning. To the extent that shared narratives govern collective behavior, narrative can facilitate the process of predicting the behavior of other members of the group, as well as making oneself more predictable to others.

Our proposal is that the engagements of an agent or group of agents with their cultural niche *through narrative practices* enable the acquisition of expectations that conform to local regimes of attention. A social niche, environment or context presents many cultural affordances for action. A pattern of shared attention conveyed by bodily, linguistic and contextual cues (patterns of eye movements, pointing gestures, etc.) guides learning, by biasing the attributions of salience of the child, and thereby conditioning specific, culturally sanctioned or normative behaviors involving when, where and how to engage with the cultural affordances of an environment (Roepstorff et al., 2010; Ramstead et al., 2016). These patterns of engagement include ways to listen to and produce stories, some of which may be ephemeral but others of which can become cognitive and social artifacts that carry much of the weight in predicting future sensory outcomes generated by causes like other person’s intentions (e.g., the intentions of the caregiver in relation to what she gestures at when guiding the child’s attention). In this way, narrative practices help the child attune to a cultural niche both through rudimentary attentional processes (e.g., attending to gaze or pointing to determine ‘What does my mother want me to look at?) and the more elaborate models that convey socially appropriate behaviors in particular contexts (e.g., ‘What do others expect of me in this situation?’), which are presumably learned mainly via implicit social learning, through active inference in earlier exposures to social situations.

In addition, active inference supports and is consistent with the MNF, which also recognizes that narratives are useful to group members as providers of predictions that guide the life course, as well as for knowledge acquisition, cooperation and social cohesion. Based on the active inference understanding that prediction is a crucial activity for adaptive organisms, we suggest that generating accurate predictions is a key role of master narratives. We argue that seeing master narratives as such can explain parsimoniously key aspects of each of the characteristics identified by McLean and Syed (2015). Furthermore, seeing narrative as a vehicle for active inference can provide answers to some pending questions about the interaction between master and alternative narratives. For example, it can help us understand why someone would go through the difficulty of adopting an alternative (and socially marginalized, contentious or disvalued) narrative instead of simply adopting master narrative (i.e., because the alternative narrative is more predictive of one’s own reality) (McLean, 2024).

In a nutshell, we contend that certain narratives ‘become’ master narratives because they serve an important function for the group. Specifically, master narratives serve to synchronize the predictions that group members generate for their own and others’ future states, behaviors, and relationships. The fact that most group members tend to adopt the same narrative models of each other’s behavior and life course (or identity) makes it easier to predict the behavior and life course of oneself and each other. When members of a culture share the same master narratives, they share a set of predictions implicitly included by this narrative; how they collectively think that events and life periods are supposed to unfold. Our model suggests that master narratives may be understood as being put in place and maintained because of their role in the synchronization of predictions in the group.

Active inference also provides a parsimonious explanation for what causes master narratives to be invisible (tacit, implicit or unconscious), ubiquitous (widely adopted by the mass of people), compulsory (normatively enforced) and rigid or persistent (hard to change). With regard to their compulsory nature, McLean and Syed (2015) hypothesize that master narratives are in place because they are enforced by those in power who benefit from the system. While this certainly has a role to play, this does not explain why many minorities tend to internalize the master narratives (which are often demeaning to them) (Nadal et al., 2021). Indeed, the counter-intuitive fact that minorities and racialized group members may automatically adopt many aspects of the master narratives that disadvantage them without necessarily having been constrained to do so is well demonstrated by research on system-justification theory (Jost and Andrews, 2011). This adoption of personally damaging master narratives is also evident in the resistance or backlash that some progressive movements face from the very people who would most benefit from the structural changes they advocate.

To account for this, as a complementary hypothesis to the power-structure explanation, we suggest that what drives group members to adopt a master narrative is partly their added ability to reduce prediction-error, which occurs independently of whether or not the individuals internalizing the narrative benefit from the system enacted or maintained by that narrative. Note that this readiness to accept a master narrative to simplify predictive processing can be leveraged by those in power to maintain the master narratives that benefit them, but we argue that one basis of master narrative internalization lies in active inference processes, which make them tacit and more or less automatic (or in the words of McLean and Syed, *invisible* and *ubiquitous*). Group members also adopt master narratives because they are a tried-and-true set of predictive models that are easily available in their social environment. In addition, some of the normative or compulsory nature of the master narratives can be understood as due to the fact that someone who presents an alternative narrative literally increases prediction-error for other group members. As a result, someone who transgresses the master narrative may experience social pressure or shaming by others to pressure them to conform in order to reduce the prediction-error that they generate for others.

In the same vein, active inference implies that master narrative change tends to be difficult and rare because it involves increasing prediction-error; this negative effect is more likely when groups share the same models (*rigidity principle*). We suggest that master narratives tend to be rigid because they have been found to be satisfactorily accurate predictions. Therefore, as McLean and Syed (2015) recognize, trying to change a master narrative by adopting an alternative narrative is difficult and may involve a risky long-term commitment. One price to pay is experiencing the consequences of being a source of prediction error for others who still adhere to the master narratives. This may require adopting a temporary position as a provocateur and agent of change, or an enduring identity as outsider who is viewed as unpredictable, disruptive, and a threat to the social order (Hyde, 1997). Nonetheless, history offers many examples of individuals and marginal groups investing in efforts to transform or replace master narratives with alternative narratives (with varying degrees of success), despite enduring the irritation or hostility of others. Active inference offers a framework to explore the tradeoffs involved in these dynamics of cognitive and social change.

The reason why master narratives are not explicitly recognized as such by the majority (*invisibility* principle) is clarified by active inference’s basic assumption that elaborate and effortful information processing is not necessary when predictions are confirmed. In effect, one reason why master narratives are ‘invisible’ to the majority is simply that they are efficient as nonconscious or implicit predictive models, and as such do need to be extensively processed or made explicit. Indeed, neuropsychological studies support the idea that predictions are more extensively processed in the event of prediction-errors (Den Ouden et al., 2012).

The MNF observes that we are strongly and automatically drawn to internalize master narratives, and that internalizing alternative narratives is uncommon and difficult. However, it raises the question: why would some individuals choose to pursue alternative narratives, if doing so is such a strenuous endeavor? We argue that casting narrative as active inference can partly answer this question with the benefit of remaining consistent with the MNF’s overall claims. Master narratives are about what is expected of us from a normative standpoint, but individuals are also able to use narrative to craft predictions that deviate from societal expectations (i.e., alternative narrative). Crucially, while it is true that internalizing an alternative narrative would generate prediction-error for other group members and thereby constitute a risk, it could also generate more predictive accuracy for its beholder, and we suggest that this is the reason why some individuals go through the ordeal of adopting them.

Relying on active inference thus explains the risks of internalizing alternative narratives (i.e., becoming the source of prediction-error for others), while simultaneously explaining why some group members would instead choose to adopt alternative narratives: namely, because the new narrative yields what more accurate predictions about events than those provided by current master narratives. Of course, applying an active inference lens to master narratives does not contradict existing work, but supplements it, notably with regard to the defining principles noted above, while offering a complementary explanation for the basic underlying principles that explain the development of master narratives.

## 4 Future directions

Our model also has implications for the overall direction of narrative research, with potential downstream consequences for several domains of psychology and the human sciences in general. For instance, while conceptualizations of narrative identity recognize that both the past and the future can contribute equally to narrative identity, a review by Syed and McLean (2016) found that almost no research on narrative identity has focused on narratives of the future. This emphasis on the past in narrative research has persisted, suggesting that there is an implicit assumption in the field that the bulk of narrative identity lies in recollecting and retelling our past, as if we were first and foremost historians of our own lives, and only then authors of its unfolding. One contribution of the active inference perspective is to shift the emphasis to consider the functions of narratives, even when they are about the past, as related to anticipating the adaptive challenges of the immediate or longer-term future. Perhaps the most relevant questions about identity then are not “Who am I?” or “Who was I?,” but “Who am I becoming?” or “Who will I be?” To examine the relative weight of past and future narratives on identity processes, future research could compare the degree to which they predict aspects of identity processes over time.

In addition, an active inference model of narrative would imply that narrative identity is chiefly about the *connection between* past and future, as active inference involves a constant back and forth between prediction-errors associated with past experience that informs predictions of the future in order to effectively navigate dynamic and surprising environments. Narratives should be especially apt to accomplish such complex interrelations between the “ongoing story” (McAdams, 2001) and a world that is constantly changing, especially given the ways that narratives of past surprises can inform narratives of the future (Philippe, 2022). Our model points to the importance of studying how narratives of the past and future are related to each other to generate possible selves and support predictions about future outcomes (Bruner, 1986).

This re-orientation is supported by the work of Chandler and Lalonde (1995) and Chandler (2000) who argued for the importance of narrative capacities in maintaining a sense of personal continuity and projecting the self into the future. Chandler developed a measure of the cognitive capacity to maintain a sense of personal continuity by presenting children with stories in which the central character underwent substantial changes in identity and asked if the character remained the same person and, if so, how they knew that. Children’s ability to warrant continuity becomes progressively more complex with cognitive development, culminating in narrative accounts that integrate the notion of continuity of identity with change through transformation. Ball and Chandler (1989) found difficulties in warranting the continuity of identity among adolescents hospitalized for suicidality compared to those admitted for eating disorders. Chandler posited that the failure to project the self into the future was a specific risk factor for suicide in the context of psychic pain, role disruption, and personal crisis (Chandler and Proulx, 2006). In related work, Chandler and Lalonde (1998, 2007) documented a relationship between community-level disruptions of collective identity and local control (which they termed “cultural continuity”) and rates of suicide among Indigenous youth across First Nations in British Columbia. This future orientation may be crucial not only for individual survival but for the survival of whole communities as Chandler has argued in relation to Indigenous Peoples (Chandler, 2013). While Chandler’s focus was on the adaptive value of imagining any possible future that is in continuity with the past, we suggest that the realizability of a predicted or imagined future is an important part of its adaptive value. The active inference approach could be used to model the interaction between collective narratives of identity and individual future-oriented identity narration.

A final conceptual contribution of our account of narratives lies in its epistemological implications. If narratives function through active inference, they can be approached in mechanistic terms to explain facets of individual and collective adaptation. Bruner’s (1986) distinction between the narrative and paradigmatic modes of knowing remains very influential. He insisted that intentionality is central to narrative, but that it plays virtually no role in paradigmatic knowing (i.e., how the physico-chemical world works). While Bruner suggested that the narrative mode of knowing pertains to aspects of the world (e.g., meaning and intentionality) that are completely distinct from those that pertain to the paradigmatic mode, a virtue of active inference modelling is that it allows us to reject the hardline distinction between the epistemological realms of human meaning and physical mechanism. Understanding narrative as active inference then provides a way to bridge the narrative and paradigmatic modes. Indeed, a constantly changing world inevitably confronts us with uncertainty and, in these instances, generating multiple narrative possibilities through imagination is useful in finding those that are a good fit. Thus, the narrative mode of knowing, which involves considering multiple possibilities, becomes a means to survive in the physical world because of the ability it affords to navigate an uncertain, imprecise, and constantly changing environment. This adaptive value of narrative imagination might be part of why we are drawn to and enjoy complex narratives with surprises, cliff-hangers, plot twists or unexpected turns: they provide us with a risk-free exploration of surprise, enlarging our repertoire of possibilities to be used in predicting events in our own lives. In sum, our active inference account of narratives suggests that Bruner’s distinction is less sharp than it might appear. Narratives are in the service of prediction and can be used to model both the physical and social world. As tools of communication and rhetorical influence, narratives are also a primary vehicle for social action in science and in everyday life.

### 4.1 Future research: the predictive validity of narratives

Studies examining predictions under the active inference framework have mostly involved the prediction of auditory (Näätänen, 1990), visual (Stagg et al., 2004), or somatosensory stimuli (Akatsuka et al., 2007), or rudimentary event sequences consisting of a succession of objects in scenes (Kumaran and Maguire, 2007). In these studies, the value of narrative lies in the capacity it affords to represent and communicate information about more complex series of events, and in doing so, increase the power to predict them in the long term. Longitudinal and experimental designs that examine the incremental value of narrative for active inference could help us better understand the mechanisms responsible for the association between narratives and adaptive functioning (e.g., Adler et al., 2016).

One research design that could be used as a basis to test our claim that narratives are related to anticipating the adaptive challenges of the immediate or longer term future is straightforward: examining the role of narrative in enhancing the accuracy of the predictions that we generate or update. D’Argembeau and Garcia Jimenez (2020) created a methodology that could be used to test hypotheses relevant to our framework. They measured the degree to which individuals could accurately predict an event that they believe could reasonably happen to them in the future. At Time 1, they asked participants to describe 10 events that they believed could happen to them in the next month. At Time 2, a month later, participants were provided with their event description and were asked if the event did or did not actually occur. We suggest that adding an experimental component to this study’s design could be used to isolate the effect of narrative on predictive accuracy. For instance, one group could provide a narrative description of prospective events, as was the case in the original study, whereas a second group could be asked to only think about the event using non-narrative means (e.g., picturing some features of the event). Researchers could use methodologies used in the affective forecast literature (Wilson and Gilbert, 2005) to create such control groups. For instance, Wilson et al. (2000) asked participants to think about a specific day, estimate what they would be doing and the number of hours they would spend on 10 activities. Here, instead of describing activities and number of hours, another group would be asked to imagine and provide a narrative description of the events. Based on our model, we hypothesize that a narrative description would be significantly more predictive of the actual occurrence of details of the narrated events in the future than would non-narrative modes of reflection. This basic framework could be extended to study the predictive power of narrative over different time scales, using longitudinal and diary study designs.

Also, approaching narrative as active inference could be leveraged to complement current studies of the links between planning and goal progress (e.g., Oettingen, 2012). Narrative has been found to bridge the divide between temporally distant events (Cohn-Sheehy et al., 2021), and thus could presumably lead to more accurate predictions of causally connected events. According to our model, using narrative to think about goal-related events in the future could facilitate goal progress because of better planning. This would result in future events that are part of planning (single events or a sequence of events leading to goal attainment) being predicted with more accuracy. This would help the individual better prepare for and take steps toward the desired outcome. In an experimental design, one could contrast two conditions (i.e., intention-only and intention-narrative), one in which a goal is intended, and another one in which goal planning is narrated (Szpunar and Szpunar, 2016). An increase in the predictive accuracy of the planning should give the narrative group an edge compared to the intention group, resulting in more goal progress.

With regard to the social function of narrative, studies could use the methodology described above to examine if master narratives (i.e., those exhibiting invisibility, ubiquity, compulsivity, and rigidity) and alternative narrative are more predictive of actual life events. The hypothesis based on active inference would be that master narratives or alternative narratives are adopted primarily because they increase predictive accuracy. Master narratives that depict a life script (Berntsen and Rubin, 2004) that is shared by members of a specific group would be especially relevant for empirical purpose. This could be tested by examining the relation of life scripts to outcomes over time. Syed and McLean (2023) provide a primer on master narrative and life script measurement useful for the design of such studies.

Future studies could reveal whether narratives can also improve the process of updating existing models to reduce prediction-error (see Habermas and Köber, 2015, for some initial evidence of this). That is, narrative use should lead to better updates of erroneous models than non-narrative means of representing errors in events. The methodology of D’Argembeau and Garcia Jimenez (2020) could be adapted to ask participants to predict an event and focus on the subgroup who fail to accurately predict the event. Those participants could then be divided into two groups. In the first group, participants would be asked to provide a narrative description of how the erroneously predicted event actually occurred, and how it should occur in the future; this would prompt belief-updating through narrative processing. In the second group, participants would only be asked to think about the erroneously predicted event and provide a single word or feature to describe it, thereby using non-narrative means of reflecting about events. Those in the narrative group would be expected to have more predictive accuracy than the other group due to the involvement of narrative in belief-updating.

The active inference model suggests that there may be different strategies for narrative updating depending on the frequency of social-environmental change. Certainly, if we had to change our models very often to understand each other, then some narratives might be more a burden than a resource. However, to the extent that individuals are encultured in the same social system or niche, they can benefit from the relative stability of socio-cultural narrative models afforded by the inclusion in the group. Overarching narratives may then provide useful predictions and guides to social adaptation. Variations in the frequency of updating may reflect the dynamics of cognitive niches and communities. There are situations where frequent updating will be taxing and others where it will foster playful creativity. The active inference framework does not currently make assumptions about the frequency of updating or its consequences *per se*, but it could be used to model and explore these cognitive and social dynamics.

## 5 Conclusion

We have proposed a framework for understanding some of the key functions of narratives, leveraging the active inference framework to clarify the cognitive and social utility of personal and event narratives. The contributions of our model to the literature include (1) an integration of the cognitive and social functions of narrative, (2) a shift towards understanding narrative as basically for predicting the future, and (3) the potential to adapt a widely used computational toolkit for modelling narrative functions. Despite its wide explanatory reach, this model is not meant to be an all-encompassing account of narrativity and the many roles narrative plays in human cognition and social interaction. In the course of human cultural coevolution, our narrative capacities have been put to many novel uses, each of which has its own scope and limits. In particular, our active inference model of narrativity does not aim to explain completely why narratives are so central to art and entertainment. The function of literary or cinematic fiction may be to help us get better at simulating human experience by widening and enriching the repertoire of models that are available to us to predict the future (Mar and Oatley, 2008). The key role of affect and emotion in these activities may reflect their role in organizing and justifying behavior in social interactions (Oatley, 1996, 1999). However, many of the aesthetic features that contribute to the appeal of narratives may be independent from their power to predict real world events. Our model also does not aim to explain why some individuals with psychological or neurological disorders may have difficulty using narrative to accurately predict their futures—though it provides a framework that could be used to develop a typology of narrative pathologies.

The current account integrates the basic functions of narrative as a representational and communicational process, through an active inference lens. Narratives segment the beginning and end of specific events, enabling the capacity to build and maintain an information pool of specific past events to inform expectations, and to structure the imagination of the future in the enriched format of episodic future projection. The meaning-making function of narrative is especially useful for the exploratory phase of active inference, in that it allows us to draw new links between various facets of our experiences to incorporate changes in representations (i.e., model updating) and assimilate unexpected information (when needed). The coherence function of narrative is used to maintain and replicate the integrity of our complex network of expectations to minimize internal contradictions, as well as providing expectations linked to culturally common situations and typical life trajectories. Narrative storytelling practices can be usefully understood via active inference, in so far as they function by providing agents with immersive regimes of attention and deontic cues that prompt encultured individuals to react in culturally prescribed ways to engage with their niche. Finally, an active inference lens clarifies the defining characteristics of master narratives, while suggesting the reasons why some individuals go through the ordeal of adopting alternative narratives that challenge the conventional story.

In sum, the importance and ubiquity of narratives in many domains of human adaptation and sociality can be parsimoniously explained by recognizing their role in human social life, via active inference. Cast as active inference, narratives are an evolved capacity that promote human adaptation for individuals and groups (McAdams, 2019). From the point of view of active inference, it becomes clear that narrative capacity is not only a medium for human cognition and sociality but through its basic structure contributes to the adaptability of individuals and communities. Active inference provides new tools for modelling the narrative dynamics that link cognition and social interaction.

## Author contributions

NB: Conceptualization, Writing – original draft, Writing – review & editing. MR: Conceptualization, Writing – original draft, Writing – review & editing. AC: Writing – review & editing, Writing – original draft. KF: Writing – review & editing. LK: Conceptualization, Writing – original draft, Writing – review & editing.
